# Brain gene expression signature on primate genomic sequence evolution

**DOI:** 10.1038/s41598-017-17462-3

**Published:** 2017-12-11

**Authors:** Shahar Barbash, Thomas P. Sakmar

**Affiliations:** 1 Laboratory of Chemical Biology and Signal Transduction, The Rockefeller University, 1230 York Ave., New York, NY 10065 USA; 2 Department of Neurobiology, Care Sciences and Society, Division for Neurogeriatrics, Center for Alzheimer Research, Karolinska Institutet, 141 57 Huddinge, Sweden

## Abstract

Considering the overwhelming changes that occurred during primate evolution in brain structure and function, one might expect corresponding changes at the molecular level. Surprisingly, a relatively constrained gene expression pattern is observed in brain compared with other tissues among primates, an observation that calls for reassessment of RNA expression influence on primate genome evolution. We built phylogenetic trees based on genomic sequences of functional genomic regions and tissue-specific RNA expression in eight tissue types for six primate species. Comparisons of the phylogenetic trees from brain tissues revealed that DNA- and RNA-based trees were significantly similar. The similarity was specific for promoter regions and cerebellum and frontal cortex expression, suggesting a major impact of gene regulation in the brain on genome shaping along the primate branch.

## Introduction

When comparing the phenotype of humans with non-human primates in the context of evolutionary fitness one might argue that the most significant changes are brain related^1^. Among these brain-related phenotypes are verbal language and advanced tool making. Indeed, the development of these brain capabilities was coupled with major structural and functional changes in the brain^2^. In recent years, researches have looked for molecular processes that could explain these human specific structural and functional distinctions^3^. Particularly, RNA expression changes among primates have been studied^4–6^. Surprisingly, among primates, gene expression in the brain is actually more constrained than gene expression in other tissues^7,8^. Given the huge changes in brain dependent phenotypes on the one hand, and the restricted changes in brain RNA expression among primates on the other, we decided to address the matter with an alternative hypothesis.

**Figure 1:**
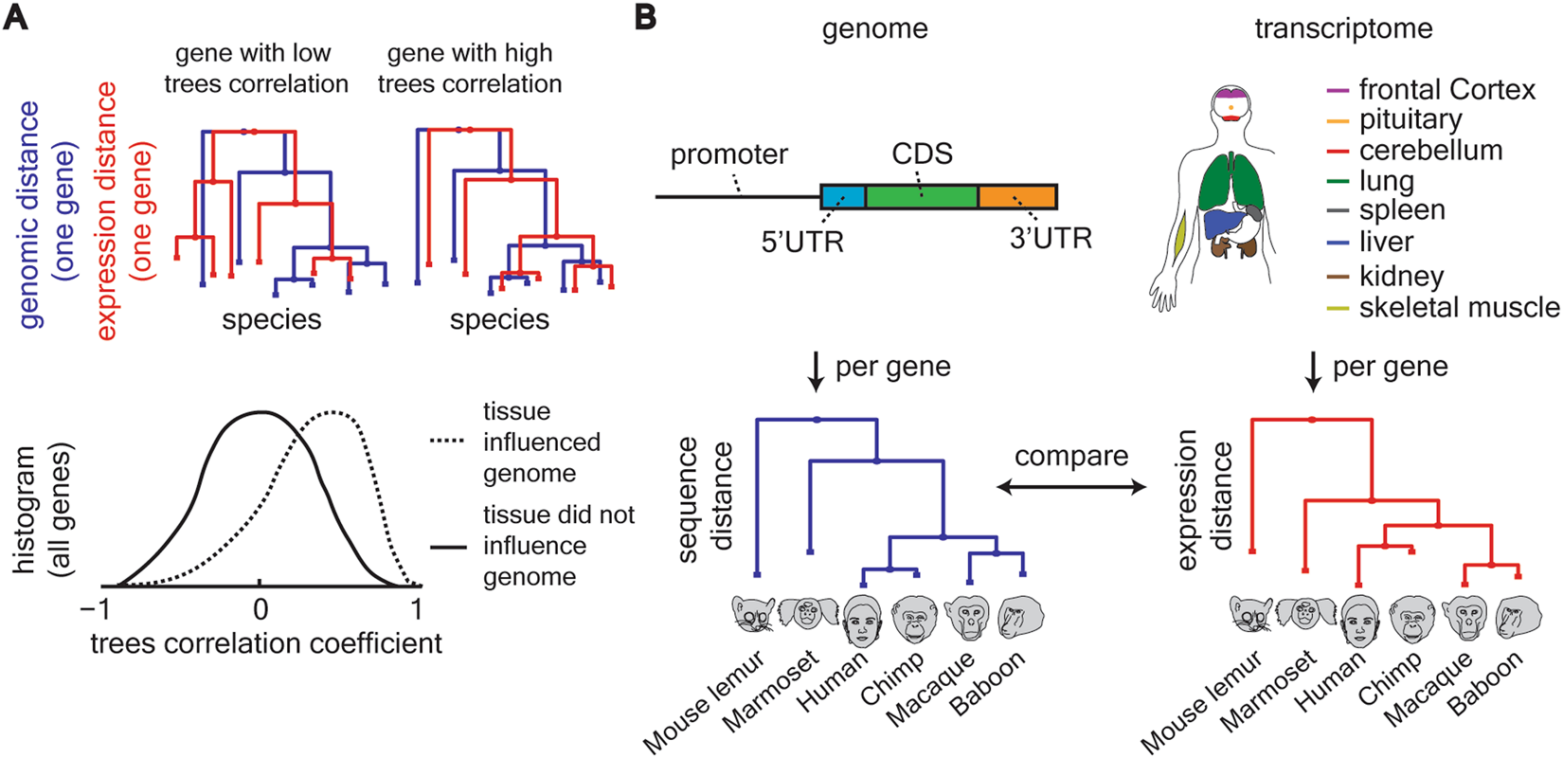
Comparing genomic sequence changes with tissue expression changes. (**A**) Genomic distance-based and expression distance-based trees were built for each genomic region and each tissue. Tree correlation was evaluated with the pair-wise Pearson correlation coefficient. At the tissue level, the distribution of the correlation coefficients for all tissue enriched genes was evaluated. A shift towards high values of correlation coefficient suggests that the tissue’s expression is associated with genomic sequence evolution. (**B**) Sequence based trees were built for six primate species (as noted above) and separately for four genomic regions. The genomic regions are 1.5 Kbp upstream of gene’s TSS, 5′UTR, CDS and 3′UTR. The upstream region was further fragmented to three equal size regions. Expression-based trees were built for the same species for eight tissues and compared against the genomic sequence trees.

We hypothesized that genomic sequence changes will be associated with expression changes in the brain more than they will with other tissues. Importantly, this hypothesis does not require large brain expression changes among primates since a particular expression change can be small in size and still highly associated with a genomic change. Therefore, while previous studies have examined the amount of brain expression change among primates, we set out to examine the strength of association between the expression change and the genomic change. A tighter association of a particular tissue expression change with a genomic change would suggest strong influence of this tissue on primate genomic evolution (Fig. 1A). Several coding and non-coding mutations that affect brain development have been previously reported^1^. The goal of our study was to estimate the global contribution of tissue-transcriptomes to genomic shaping and compare these estimates among genomic regions and tissues in a systematic and unbiased manner.

## Results

To evaluate the hypothesis that genomic sequence changes will be associated with expression changes particularly in the brain, we built and compared phylogenetic trees based on sequence distance, and separately based on tissue expression distance. These expression- and genomic-based trees were built using expression and genomic data from six primates and included eight tissues for each species (Fig. 1B). The six examined species represent five major primate groups: Humans (human), Great Apes (chimpanzee), Old World Monkeys (baboon and macaque), New World Monkeys (marmoset) and Prosimians (mouse lemur). Mutations in an organism’s DNA could lead to a change in protein structure or a change in the temporal and spatial pattern of the gene’s expression^9^. Both of these outcomes, namely changes in protein structure and gene expression, are believed to have phenotypic effects on primate evolution^10^. Therefore, the genomic trees were separately built for four distinct functional genomic regions, including coding and non-coding regions (Fig. 1B).

In order to build genomic-based trees, we downloaded the genomes of six primates (human, chimpanzee, rhesus macaque, olive baboon, common marmoset and mouse lemur) from Ensemble Genome Browser. In order to build expression-based trees, we downloaded tissue expression data from The Nonhuman Primate Reference Transcriptome Resource (NHPRTR)^11^ and additional human expression data from The Genotype-Tissue Expression (GTEx) project^12^ (see Methods for full details on analyzed data). From the genomic data we collected sequences of the following genomic regions for each gene: promotor region of 1.5 Kbp upstream to gene’s transcription start site (TSS, similar to a previous study^8^), 5′ untranslated region (5′ UTR), coding sequence (CDS) and 3′ untranslated region (3′UTR; see Methods for regions‘ definition). The promotor region was further fragmented into three equal-size windows: from 1.5 Kbp to 1 Kbp, from 1 Kbp to 0.5 Kbp and from 0.5 Kbp to 0 Kbp upstream to TSS (see Fig. S1 for regions‘ length analysis). In addition, a region of 1.5 Kbp upstream of the promoter region was analyzed. We observed longer 3′UTRs for humans compare to non-human primates recapitulating previous reports by others^13^. Next, for each gene, we built a separate phylogenetic tree based on each genomic region. We completely filtered out any region that contained undefined nucleotides (‘N’ in sequence) since these introduce artifacts into phylogenetic analysis. To account for general inter-species genomic distances, each phylogenetic gene tree was normalized by the global genomic tree (% difference, Fig. S2). The normalizing tree was downloaded from http://www.gate.net/~rwms/primegendist.html#Msp where the distances between the nodes were calculated by the PAUP*4.0 software. Because different organisms and tissues have different expression distributions, all expression data were quantile-normalized^14^ (see Methods and Fig. S1). This procedure produces comparable expression distributions of identical statistical properties (Fig. S3).

**Figure 2:**
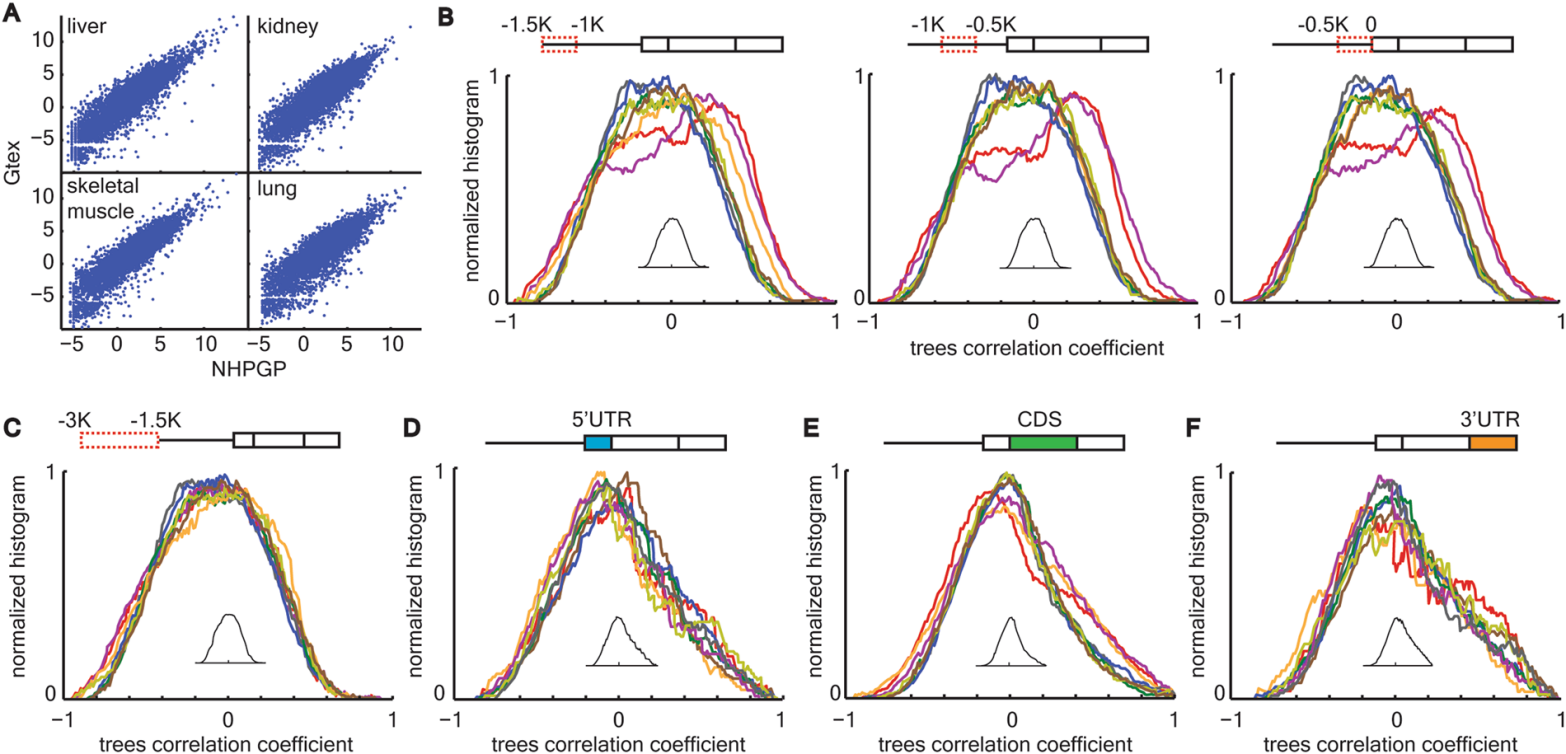
High correlations between promoter region evolution and brain expression. (**A**) High correlation between NHPRTR and GTEx expression data. Scatter plot comparing between quantile normalized expression values for the same human tissue for liver, kidney, skeletal muscle and lung, from the NHPRTR database (X axis; log2 transformed) and the GTEx database (Y axis; log2 transformed). Pearson correlation r > 0.987 in all four comparisons. (**B**) Transcriptome tissue color codes are shown in Fig. 1B. Correlation coefficient distributions for three consecutive 500 bp windows in the promoter region show significant shifts towards high values in cerebellum and frontal cortex. Kolmogorov Smirnov P of individual distributions versus the bootstrap distributions (shown as inset) = 2*10^–4^, P = 4*10^–7^ and P = 6*10^–6^ for left, middle and right windows, correspondingly, for frontal cortex and P = 3*10^–3^, P = 3*10^–6^ and P = 5*10^–6^ for left, middle and right windows, correspondingly, for cerebellum. P > 0.05 for or all other comparisons. (**C**) Transcriptome tissue color codes are shown in Fig. 1B. Correlation coefficient distributions for 3 Kbp to 1.5 Kbp upstream of transcription start site did not show any shift compared with the bootstrap distribution. P > 0.05 for all comparisons. (**D-F**) Transcriptome tissue color codes are shown in Fig. 1B. Correlation coefficient distributions for 5′UTR (**D**), CDS (**E**) and 3′UTR (**F**) did not show any shift compared with the bootstrap distribution. P > 0.05 for all comparisons. P values are Bonferroni corrected.

Several tissues are represented in the NHPRTR database for non-human primates, but not for humans. Data for corresponding tissues from humans were hence downloaded from the GTEx database. To verify that the GTEx and NHPRTR databases are comparable, we analyzed four transcriptomes of human tissues that existed in both databases. We observed extremely high expression correlations for each of the four examined tissues (Fig. 2A). The total number of genes identified in all examined primates RNA expression data was 26,204. Based on the quantile normalized expression values, we separately built Euclidean distance trees for each gene in each tissue across organisms. In these expression-based trees the distance between any given pair of species equals the delta between their quantile-normalized expression (see Methods). Previous studies showed shorter expression distance between primates based on brain transcriptome compared to non-neuronal tissues^7,8^. Calculating the total expression-based tree across organisms for each tissue, we recapitulated this result (Fig. S4A). For orthologous genes, we used Ensemble annotation of ‘one-2-one’ orthology between primates. The number of orthologous protein coding genes without ‘N’ in their genomic sequence in any of the primate genomes was 4812 for up-stream of promoter region, 4866 for promoter region, 6931 for 5′UTR, 6604 for CDS and 6873 for 3′UTR.

Our hypothesis suggests that beneficial phenotypes that depend on gene expression patterns were consolidated and represented by changes in genomic shaping around these genes. In order for an expression change to be passed to the offspring it is likely to be manifested as a genomic change. The alternative that changes in expression pattern could be due to epigenetic alteration (*i*.*e*., transgenerational epigenetic inheritance)^15^ is not addressed in this paper. Therefore, regions or genes that were important for evolutionary genomic sequence consolidation in the organism would have similar expression-based and genomic-based trees. We searched for trees similarity by calculating the Pearson correlation coefficient, pair-wise, between expression-based and genomic-based trees (see comment in Methods about additional ways to compare trees). Genes with low trees similarity would have low correlation coefficient while genes with high trees similarity would have high correlation coefficient (Fig. 1A). To assess the contribution of a tissue to genomic sequence consolidation, we examined the shape of the distribution for the calculated correlation coefficient for tissue-enriched genes (see definition below). Our hypothesis predicts that tissues that have a weak influence on genomic sequence consolidation would have a correlation coefficient distribution concentrated around zero (solid line in Fig. 1A). On the other hand, tissues that have strong influence on genomic sequence consolidation would have distributions shifted towards positive values (dashed line in Fig. 1A).

To examine this hypothesis on a tissue by tissue level, we first defined enriched gene sets for each tissue. Tissue enriched gene sets were defined as genes expressed in a particular tissue at least five times higher than the average across all other tissues. Among the 15 examined tissues, only eight tissues showed more than 100 tissue enriched genes, which was set as the lower threshold for the number of genes. Setting a threshold on the number of tissue expressed genes is critical for assessing tissue effects instead of effects driven by only few genes. This threshold yielded between 232 and 665 genes for the eight tissues that passed the threshold. For each genomic region, we calculated and plotted the distribution of correlation coefficients between its sequence-based trees and tissue expression trees for the corresponding tissue enriched genes (Fig. 2B–D). Since there were six genomic regions and eight tissues in total, a total of 48 distributions were calculated. Because of the different number of genes in each distribution, and in order for them to be comparable these distributions were normalized by their total area underneath the curve.

In order to calculate statistical significance effects between tissues in each genomic region, we took a bootstrap approach. In the bootstrap analysis we permuted across gene trees for each tissue such that expression-based and genomic-based trees were randomly paired and a correlation coefficient was calculated. This permutation was repeated for 1000 iterations and averaged across the iterations. The outcome of this bootstrap strategy is a null distribution of correlation coefficients per genomic region, per tissue. Because tissues showed almost identical null distributions we averaged them together. Next, the actual distribution of a tissue and genomic region was compared with the bootstrap distribution by two statistical tests for comparing distributions (Kolmogorov Smirnov and Kruskal Wallis tests). *P* values were corrected with Bonferroni for multiple comparisons. Among all examined genomic regions and tissues only promoter regions in cerebellum and frontal cortex showed significant shifts towards positive correlation coefficients (Fig. 2B). This effect was significant in all three sub-regions (windows of 0.5 Kbp upstream of TSS) of the promoter region but was most pronounced in the middle window (1 Kbp to 0.5 Kbp upstream of TSS, Fig. 2B). Supplementary Tables 1 and 2 show the top 50 genomic signature genes based on cerebellum and frontal cortex expression, correspondingly. None of the other genomic regions showed any such effect for any of the examined tissues, including cerebellum and frontal cortex (Fig. 2C-F).

## Discussion

The similarity between expression-based and genomic-based trees in cerebellum and frontal cortex was higher than among all other examined tissues and higher than that predicted by chance. These results point at a detectable signature of RNA expression change in brain tissues on primates‘ genomic evolution. To the best of our knowledge, these results are the first molecular level, transcriptome-wide supportive evidence for a significant impact of the brain on genomic sequence consolidation during primate evolution.

It was previously proposed that regulatory elements, other than the promoter region, played a role in genomic shaping as well^16^. Some of these regulatory elements are 3′UTR and 5′UTR, which we have examined in our study. Our results do not contradict this hypothesis; rather, they show that the association between genomic changes and tissue expression changes is most robust and widespread around promoter regions and for brain tissues. It is possible that a similar effect acted on 3′UTRs and 5′UTRs during evolution but was too subtle to leave a detectable signature in living primates in present time. In addition, previous studies showed a pivotal role in tissue-specific gene expression for predicted enhancer regions during development in mice^17,18^. At present, no corresponding data are available to predict enhancers for the non-human primates in the tissues examined here. Therefore, these important genomic regions could not be analyzed in the same manner as the regions in our study. However, it is possible that comparisons of genomic- and transcriptomic-based trees across genomic enhancers would show a stronger signal than the reported one for promoter regions.

## Methods

### Analyzed data

In this study we analyzed genomes and transcriptomes from six species. For each species, following are the organism specific name/taxon ID/Ensembl assemblies. Human, Homo sapiens/9606/GRCh38.p7. Chimpanzee, Pan troglodytes/9598/CHIMP2.1.4. Macaque, Macaca mulatta/9544/Mmul_8.0.1. Olive baboon, Papio Anubis/9555/PapAnu2.0. Marmoset, Callithrix jacchus/9483/C_jacchus3.2.1. Mouse Lemur, Microcebus murinus/30608/Mmur_2.0. The Nonhuman Primate Reference Transcriptome Resource (NHPRTR) contains RNAseq analysis of tissue pools for individual NHP species and its full data are available on http://nhprtr.org/. The Genotype-Tissue Expression (GTEx) project collects genomic variation and tissue expression data in human population. In this study we utilized only expression data from the GTEx resource. Data available on the GTEx Portal (http://www.gtexportal.org/home/). Data from all resources were downloaded during March 2017.

### Genomic regions

We have used Ensembl annotation to define start and end sites of distinct genomic regions. The examined genomic regions were promoter region, 5′ untranslated region (5′UTR), coding sequence region (CDS) and 3′ untranslated region (3′UTR). 5′UTR was defined as the region between transcription start site to translation start site and 3′UTR was defined as the region between translation end site to transcription end site. CDS was the collection of gene’s exons. Genomic regions were collected for all existing variants per gene. For genes with multiple variants we unified regions of the same type. We followed a previous study in defining the limit for promoter region^8^. Promoter region upstream limit was set to 1.5 Kbp upstream of transcription start site. Because of the somewhat arbitrary nature of this limit we further fragmented it to three consecutive widows of 500 bp each and analyzed the sequences for each sub-region separately.

### Tree building

Genomic trees. For each gene, in each species pair, and based on each genomic region separately, a sequence distance was calculated as the proportion of sites at which two sequences are different; *p*. *p* is close to 1 for poorly related sequences and close to 0 for similar sequences. Next, *p* was transformed to Jukes-Cantor Maximum likelihood estimate of the number of substitutions between two sequences, *d*, as shown in equation (1).

1 $$d=-\frac{3}{4}\,\mathrm{log}(1-p\,\ast \,\frac{4}{3})$$

Trees were built based on *d*, such that the distance in the tree between any pair of species would be *d*.

Expression trees. For each gene, in each species pair, and based on expression of each tissue separately, we calculated the difference (delta) between their quantile normalized expression. Trees were built based on delta, such that the distance in the tree between any pair of species would be delta.

### Tree comparison

The similarity between sequence- and expression-based trees was evaluated by the Pearson correlation coefficient in a species pairwise manner. Evaluating trees‘ similarity with Pearson correlation coefficient is based on the fact that for similar trees, if the relative distance between a pair of species is high in one tree it is expected to be high in the other and vice versa. This pattern is accurately captured by the Pearson correlation coefficient and so pairwise correlation indicates similar tree structure. An alternative way to compare phylogenetic trees is the phylo-comparison algorithm^19^ which compares species partitioning between trees. This method is not sensitive for the relative distances between species inside a tree but only to species grouping on tree branches. We have not used this method due to the relatively low number of analyzed organisms. In this algorithm, low number of analyzed organisms causes the possible output scores to be restricted to just a few values. This quantized nature of the outcome makes it impossible to identify a distribution shift. For that reason we worked with the pairwise correlation between normalized trees.

### Bootstrap

In order to calculate statistical significance for the observed distribution shifts we took a bootstrap approach. We permuted across genes so that the calculated correlation coefficients were between sequence tree of one gene and expression tree of another. This gene permutation was performed for each tissue separately, to build the tissue null distribution. Because different tissues showed almost identical null distributions we averaged these distributions. This gave rise to the bootstrap null distribution, averaged across tissues, for each genomic region separately (shown as insets in Fig. 2B–F). Lastly, the correlation coefficient distribution for each tissue and each genomic region was compared to the bootstrap null distribution with statistical tests for distribution comparison, namely, Kolmogorov Smirnov and Kruskal Wallis tests, and P values were corrected for multiple comparisons with the Bonferroni correction.

### Data availability

NHPRTR full data are available at http://nhprtr.org/. GTEx full data are available at http://www.gtexportal.org/home/.

## Electronic supplementary material

Supplementary Tables and Figures
