# Supplementary material for: Brain gene expression signature on primate genomic sequence evolution
Source: Sci Rep. 2017 Dec 11;7:17329. doi: 10.1038/s41598-017-17462-3 (PMC5725417; doi:10.1038/s41598-017-17462-3)
Supplement: Supplementary file 1 — Supplementary Tables and Figures [file 41598_2017_17462_MOESM1_ESM.pdf]

Supplementary Materials for

Brain gene expression signature on primate genomic sequence evolution

Shahar Barbash and Thomas Paul Sakmar

Correspondence to: sakmar@rockefeller.edu

Supplementary Tables

Supplementary Table 1: Fifty top genomic signature genes based on cerebellum expression. Official gene symbols are reported together with Ensembl gene annotation across the six examined primates.

Gene symbol	Human	Chimp	Marmoset	Mouse Lemur	Babbon	Macaque
**MAP3K12**	ENSG00000139625	ENSPTRG00000005019	ENSCJAG00000018634	ENSMICG00000011662	ENSPANG00000020580	ENSMMUG00000006613
**SLC17A6**	ENSG00000091664	ENSPTRG00000003444	ENSCJAG00000006408	ENSMICG00000038594	ENSPANG00000015329	ENSMMUG00000020368
**CDH22**	ENSG00000149654	ENSPTRG00000013581	ENSCJAG00000017106	ENSMICG00000029501	ENSPANG00000026139	ENSMMUG00000004479
**INPP5J**	ENSG00000185133	ENSPTRG00000014262	ENSCJAG00000010779	ENSMICG00000008559	ENSPANG00000018617	ENSMMUG00000022395
**CNTNAP1**	ENSG00000108797	ENSPTRG00000009216	ENSCJAG00000013408	ENSMICG00000010313	ENSPANG00000021426	ENSMMUG00000006091
**TFAP2E**	ENSG00000116819	ENSPTRG00000000523	ENSCJAG00000001531	ENSMICG00000030619	ENSPANG00000004801	ENSMMUG00000019815
**DNASE1L2**	ENSG00000167968	ENSPTRG00000007643	ENSCJAG00000014997	ENSMICG00000005898	ENSPANG00000006417	ENSMMUG00000019236
**C1QTNF8**	ENSG00000184471	ENSPTRG00000007577	ENSCJAG00000002657	ENSMICG00000005932	ENSPANG00000023388	ENSMMUG00000001688
**GRM4**	ENSG00000124493	ENSPTRG00000018059	ENSCJAG00000015287	ENSMICG00000003131	ENSPANG00000012328	ENSMMUG00000023046
**ZNF653**	ENSG00000161914	ENSPTRG00000010507	ENSCJAG00000005551	ENSMICG00000034400	ENSPANG00000016446	ENSMMUG00000020617
**CACNA1B**	ENSG00000148408	ENSPTRG00000021608	ENSCJAG00000013488	ENSMICG00000016743	ENSPANG00000026439	ENSMMUG00000009187
**GRIN2C**	ENSG00000161509	ENSPTRG00000009619	ENSCJAG00000014754	ENSMICG00000010565	ENSPANG00000020268	ENSMMUG00000005744
**NDST4**	ENSG00000138653	ENSPTRG00000016386	ENSCJAG00000003443	ENSMICG00000033837	ENSPANG00000008604	ENSMMUG00000002883
**ST18**	ENSG00000147488	ENSPTRG00000020245	ENSCJAG00000014630	ENSMICG00000003705	ENSPANG00000009233	ENSMMUG00000008439
**SPTBN2**	ENSG00000173898	ENSPTRG00000003936	ENSCJAG00000001228	ENSMICG00000003823	ENSPANG00000011003	ENSMMUG00000020359

**WSCD2**	ENSG00000075035	ENSPTRG00000005402	ENSCJAG00000015311	ENSMICG00000014280	ENSPANG00000016135	ENSMMUG00000019042
**CADM3**	ENSG00000162706	ENSPTRG00000001520	ENSCJAG00000006307	ENSMICG00000011231	ENSPANG00000019563	ENSMMUG00000001297
**BSN**	ENSG00000164061	ENSPTRG00000014927	ENSCJAG00000004003	ENSMICG00000010621	ENSPANG00000010033	ENSMMUG00000014537
**NEUROD2**	ENSG00000171532	ENSPTRG00000009094	ENSCJAG00000008704	ENSMICG00000035050	ENSPANG00000021818	ENSMMUG00000001404
**GBX2**	ENSG00000168505	ENSPTRG00000013068	ENSCJAG00000004419	ENSMICG00000028176	ENSPANG00000017536	ENSMMUG00000005911
**SSTR2**	ENSG00000180616	ENSPTRG00000009594	ENSCJAG00000019937	ENSMICG00000031045	ENSPANG00000020325	ENSMMUG00000020947
**RTN4R**	ENSG00000040608	ENSPTRG00000014079	ENSCJAG00000010718	ENSMICG00000027043	ENSPANG00000019636	ENSMMUG00000011547
**SYT12**	ENSG00000173227	ENSPTRG00000003943	ENSCJAG00000001134	ENSMICG00000015472	ENSPANG00000004416	ENSMMUG00000011741
**KIAA1984**	ENSG00000213213	ENSPTRG00000021573	ENSCJAG00000011036	ENSMICG00000031589	ENSPANG00000026166	ENSMMUG00000005040
**TMEM63C**	ENSG00000165548	ENSPTRG00000006574	ENSCJAG00000016712	ENSMICG00000017135	ENSPANG00000021770	ENSMMUG00000001092
**WDR49**	ENSG00000174776	ENSPTRG00000015597	ENSCJAG00000002774	ENSMICG00000026583	ENSPANG00000008048	ENSMMUG00000022925
**TTBK1**	ENSG00000146216	ENSPTRG00000018193	ENSCJAG00000005343	ENSMICG00000008369	ENSPANG00000021604	ENSMMUG00000017322
**CDH20**	ENSG00000101542	ENSPTRG00000010069	ENSCJAG00000003409	ENSMICG00000032447	ENSPANG00000003495	ENSMMUG00000001713
**SBK1**	ENSG00000188322	ENSPTRG00000007921	ENSCJAG00000006098	ENSMICG00000001939	ENSPANG00000017104	ENSMMUG00000000515
**MIXL1**	ENSG00000185155	ENSPTRG00000002047	ENSCJAG00000012740	ENSMICG00000027230	ENSPANG00000007593	ENSMMUG00000005129
**VAT1L**	ENSG00000171724	ENSPTRG00000008374	ENSCJAG00000014046	ENSMICG00000009243	ENSPANG00000018199	ENSMMUG00000011797
**TMEM229B**	ENSG00000198133	ENSPTRG00000030714	ENSCJAG00000015358	ENSMICG00000037311	ENSPANG00000005728	ENSMMUG00000048801
**TRIM62**	ENSG00000116525	ENSPTRG00000000502	ENSCJAG00000001177	ENSMICG00000028440	ENSPANG00000015281	ENSMMUG00000015097
**LURAP1**	ENSG00000171357	ENSPTRG00000000688	ENSCJAG00000021078	ENSMICG00000003264	ENSPANG00000006505	ENSMMUG00000005838
**ABCG4**	ENSG00000172350	ENSPTRG00000004373	ENSCJAG00000013965	ENSMICG00000002738	ENSPANG00000004725	ENSMMUG00000013799
**PLP1**	ENSG00000123560	ENSPTRG00000022133	ENSCJAG00000003349	ENSMICG00000003053	ENSPANG00000025091	ENSMMUG00000000809
**SCRG1**	ENSG00000164106	ENSPTRG00000016599	ENSCJAG00000005138	ENSMICG00000008612	ENSPANG00000011779	ENSMMUG00000002399
**SLC5A11**	ENSG00000158865	ENSPTRG00000007899	ENSCJAG00000020690	ENSMICG00000005905	ENSPANG00000017009	ENSMMUG00000001407
**MAST1**	ENSG00000105613	ENSPTRG00000010545	ENSCJAG00000004772	ENSMICG00000016221	ENSPANG00000015630	ENSMMUG00000008740
**HTR5A**	ENSG00000157219	ENSPTRG00000019904	ENSCJAG00000000158	ENSMICG00000030603	ENSPANG00000021639	ENSMMUG00000013084

**AQP4**	ENSG00000 171885	ENSPTRG000 00009936	ENSCJAG0000 0020734	ENSMICG000 00002328	ENSPANG000 00024708	ENSMMUG000 00014934
**ANLN**	ENSG00000 011426	ENSPTRG000 00019084	ENSCJAG0000 0012416	ENSMICG000 00008936	ENSPANG000 00006301	ENSMMUG000 00021186
**TMEM178A**	ENSG00000 152154	ENSPTRG000 00011859	ENSCJAG0000 0014915	ENSMICG000 00008025	ENSPANG000 00022194	ENSMMUG000 00043458
**GLRA2**	ENSG00000 101958	ENSPTRG000 00021679	ENSCJAG0000 0004058	ENSMICG000 00015578	ENSPANG000 00003157	ENSMMUG000 00003482
**CACNG7**	ENSG00000 105605	ENSPTRG000 00011436	ENSCJAG0000 0018478	ENSMICG000 00027690	ENSPANG000 00025512	ENSMMUG000 00007975
**CHRNA2**	ENSG00000 120903	ENSPTRG000 00020105	ENSCJAG0000 0019690	ENSMICG000 00007702	ENSPANG000 00015733	ENSMMUG000 00021514
**ACSBG1**	ENSG00000 103740	ENSPTRG000 00007337	ENSCJAG0000 0010176	ENSMICG000 00008772	ENSPANG000 00015826	ENSMMUG000 00011839
**MAB21L1**	ENSG00000 180660	ENSPTRG000 00005777	ENSCJAG0000 0022569	ENSMICG000 00031296	ENSPANG000 00011126	ENSMMUG000 00005425
**NMNAT2**	ENSG00000 157064	ENSPTRG000 00001763	ENSCJAG0000 0005105	ENSMICG000 00007134	ENSPANG000 00011948	ENSMMUG000 00001481
**SNAP25**	ENSG00000 132639	ENSPTRG000 00013246	ENSCJAG0000 0021350	ENSMICG000 00003556	ENSPANG000 00003900	ENSMMUG000 00011064

Supplementary Table 2: Fifty top genomic signature genes based on frontal cortex expression. Official gene symbols are reported together with Ensembl gene annotation across the six examined primates.

Gene symbol	Human	Chimp	Marmoset	Mouse Lemur	Babbon	Macaque
**SNTG1**	ENSG0000 0147481	ENSPTRG000 00020240	ENSCJAG000 00015678	ENSMICG000 00001818	ENSPANG000 00017058	ENSMMUG00 000002811
**BAI1**	ENSG0000 0181790	ENSPTRG000 00020635	ENSCJAG000 00010346	ENSMICG000 00015976	ENSPANG000 00015392	ENSMMUG00 000019877
**RALYL**	ENSG0000 0184672	ENSPTRG000 00020381	ENSCJAG000 00000402	ENSMICG000 00005558	ENSPANG000 00019755	ENSMMUG00 000007115
**KCNF1**	ENSG0000 0162975	ENSPTRG000 00030563	ENSCJAG000 00022930	ENSMICG000 00017370	ENSPANG000 00015920	ENSMMUG00 000002825
**EFR3B**	ENSG0000 0084710	ENSPTRG000 00011720	ENSCJAG000 00008127	ENSMICG000 00001606	ENSPANG000 00004979	ENSMMUG00 000016461
**HES5**	ENSG0000 0197921	ENSPTRG000 00024233	ENSCJAG000 00002549	ENSMICG000 00038417	ENSPANG000 00022730	ENSMMUG00 000044043
**PRKCG**	ENSG0000 0126583	ENSPTRG000 00011435	ENSCJAG000 00018393	ENSMICG000 00015697	ENSPANG000 00015248	ENSMMUG00 000007972
**NLGN3**	ENSG0000 0196338	ENSPTRG000 00022007	ENSCJAG000 00013566	ENSMICG000 00014073	ENSPANG000 00015903	ENSMMUG00 000032427
**OLIG2**	ENSG0000 0205927	ENSPTRG000 00013863	ENSCJAG000 00009439	ENSMICG000 00031221	ENSPANG000 00007664	ENSMMUG00 000017652
**RTN4RL 1**	ENSG0000 0185924	ENSPTRG000 00008541	ENSCJAG000 00001844	ENSMICG000 00012885	ENSPANG000 00001706	ENSMMUG00 000046616

**NANOS3**	ENSG00000187556	ENSPTRG00000010568	ENSCJAG00000004388	ENSMICG00000030950	ENSPANG00000015037	ENSMMUG00000008316
**UNC5A**	ENSG00000113763	ENSPTRG00000017569	ENSCJAG00000007237	ENSMICG00000033115	ENSPANG00000011858	ENSMMUG00000002649
**SPRED3**	ENSG00000188766	ENSPTRG00000010928	ENSCJAG00000013614	ENSMICG00000027687	ENSPANG00000006214	ENSMMUG00000022975
**GAD1**	ENSG00000128683	ENSPTRG00000012626	ENSCJAG00000006984	ENSMICG00000015011	ENSPANG00000024165	ENSMMUG00000015198
**GABRA4**	ENSG00000109158	ENSPTRG00000016029	ENSCJAG00000018992	ENSMICG00000017780	ENSPANG00000014209	ENSMMUG00000009526
**NPY**	ENSG00000122585	ENSPTRG00000018992	ENSCJAG00000007671	ENSMICG00000000511	ENSPANG00000021597	ENSMMUG00000009818
**CLEC2L**	ENSG00000236279	ENSPTRG00000039273	ENSCJAG00000013258	ENSMICG00000036417	ENSPANG00000004966	ENSMMUG00000037714
**RGS7BP**	ENSG00000186479	ENSPTRG00000016921	ENSCJAG00000015655	ENSMICG00000030250	ENSPANG00000016503	ENSMMUG00000021131
**STMN3**	ENSG00000197457	ENSPTRG00000030476	ENSCJAG00000010324	ENSMICG00000029780	ENSPANG00000024130	ENSMMUG00000021297
**NDP**	ENSG00000124479	ENSPTRG00000021818	ENSCJAG00000010835	ENSMICG00000034006	ENSPANG00000016329	ENSMMUG00000018001
**LPPR4**	ENSG00000117600	ENSPTRG00000000994	ENSCJAG00000018252	ENSMICG00000032022	ENSPANG00000006691	ENSMMUG00000021030
**CACNG3**	ENSG00000006116	ENSPTRG00000007896	ENSCJAG00000020656	ENSMICG00000008055	ENSPANG00000023573	ENSMMUG00000013560
**TRIM9**	ENSG00000100505	ENSPTRG00000006338	ENSCJAG00000006884	ENSMICG00000013634	ENSPANG00000025018	ENSMMUG00000011051
**MAST1**	ENSG00000105613	ENSPTRG00000010545	ENSCJAG00000004772	ENSMICG00000016221	ENSPANG00000015630	ENSMMUG00000008740
**C1QTNF4**	ENSG00000172247	ENSPTRG00000003572	ENSCJAG00000020211	ENSMICG00000027804	ENSPANG00000000111	ENSMMUG00000004294
**CCDC85A**	ENSG00000055813	ENSPTRG00000011940	ENSCJAG00000009663	ENSMICG00000029777	ENSPANG00000016914	ENSMMUG00000015888
**LINGO2**	ENSG00000174482	ENSPTRG00000020840	ENSCJAG00000022711	ENSMICG00000004873	ENSPANG00000015960	ENSMMUG00000017199
**ATL1**	ENSG00000198513	ENSPTRG00000023829	ENSCJAG00000006480	ENSMICG00000005443	ENSPANG00000014239	ENSMMUG00000009577
**MARCH4**	ENSG00000144583	ENSPTRG00000012897	ENSCJAG00000012374	ENSMICG00000027749	ENSPANG00000022150	ENSMMUG00000020824
**CDH9**	ENSG00000113100	ENSPTRG00000016759	ENSCJAG00000007662	ENSMICG00000008404	ENSPANG00000012091	ENSMMUG00000019846
**SV2B**	ENSG00000185518	ENSPTRG00000007469	ENSCJAG00000019298	ENSMICG00000017792	ENSPANG00000014252	ENSMMUG00000008399
**RORB**	ENSG00000198963	ENSPTRG00000021025	ENSCJAG00000001216	ENSMICG00000007512	ENSPANG00000024927	ENSMMUG00000012571
**PSD2**	ENSG00000146005	ENSPTRG00000017305	ENSCJAG00000016848	ENSMICG00000012647	ENSPANG00000002492	ENSMMUG00000014636

**HMGCLL1**	ENSG00000146151	ENSPTRG00000018299	ENSCJAG00000010832	ENSMICG00000015183	ENSPANG00000000755	ENSMMUG00000001399
**MAP3K19**	ENSG00000176601	ENSPTRG00000012490	ENSCJAG00000011631	ENSMICG00000013841	ENSPANG00000000669	ENSMMUG00000017121
**PDXP**	ENSG00000241360	ENSPTRG00000041879	ENSCJAG00000012519	ENSMICG00000003819	ENSPANG00000017416	ENSMMUG00000049216
**KCNK4**	ENSG00000182450	ENSPTRG00000003832	ENSCJAG00000018060	ENSMICG00000016981	ENSPANG00000018628	ENSMMUG00000017802
**BSN**	ENSG00000164061	ENSPTRG00000014927	ENSCJAG00000004003	ENSMICG00000010621	ENSPANG00000010033	ENSMMUG00000014537
**SLC2A13**	ENSG00000151229	ENSPTRG00000004827	ENSCJAG00000017922	ENSMICG00000034052	ENSPANG00000019087	ENSMMUG00000010078
**KCNIP3**	ENSG00000115041	ENSPTRG00000012213	ENSCJAG00000018883	ENSMICG00000010744	ENSPANG00000018833	ENSMMUG00000000199
**TTBK1**	ENSG00000146216	ENSPTRG00000018193	ENSCJAG00000005343	ENSMICG00000008369	ENSPANG00000021604	ENSMMUG00000017322
**CACNG7**	ENSG00000105605	ENSPTRG00000011436	ENSCJAG00000018478	ENSMICG00000027690	ENSPANG00000025512	ENSMMUG00000007975
**CADM2**	ENSG00000175161	ENSPTRG00000015120	ENSCJAG00000008885	ENSMICG00000014503	ENSPANG00000006775	ENSMMUG00000000583
**GPR151**	ENSG00000173250	ENSPTRG00000017376	ENSCJAG00000021711	ENSMICG00000009365	ENSPANG00000016847	ENSMMUG00000023287
**SLC30A3**	ENSG00000115194	ENSPTRG00000011757	ENSCJAG00000001114	ENSMICG00000017103	ENSPANG00000016958	ENSMMUG00000010272
**RAB3A**	ENSG00000105649	ENSPTRG00000010697	ENSCJAG00000014111	ENSMICG00000014724	ENSPANG00000009376	ENSMMUG00000005027
**FSD1**	ENSG00000105255	ENSPTRG00000010302	ENSCJAG00000014650	ENSMICG00000000316	ENSPANG00000019255	ENSMMUG00000000268
**MGAT4C**	ENSG00000182050	ENSPTRG00000005275	ENSCJAG00000019762	ENSMICG00000037297	ENSPANG00000014454	ENSMMUG00000023103
**RPRM**	ENSG00000177519	ENSPTRG00000012545	ENSCJAG00000023133	ENSMICG00000038767	ENSPANG00000016888	ENSMMUG00000022529
**SST**	ENSG00000157005	ENSPTRG00000015727	ENSCJAG00000014415	ENSMICG00000036921	ENSPANG00000015209	ENSMMUG00000000850

Supplementary Figures

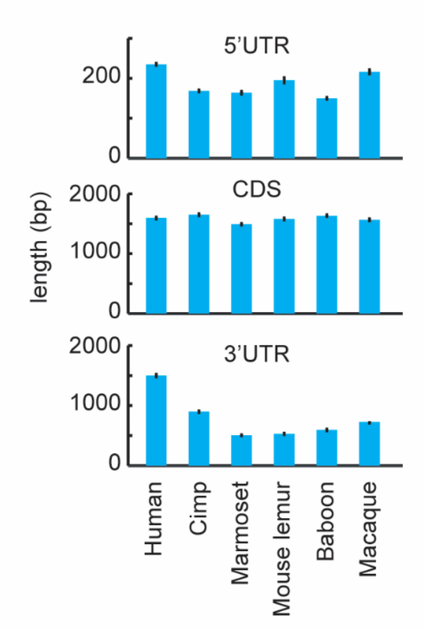

Fig. S1. Length analysis of genomic regions. Bar graph showing average and standard error of the mean (s.e.m) for lengths of the examined genomic regions: 5'UTR, CDS and 3'UTR, across the 6 examined primates. The significantly longer length of human 3'UTR (One-Way-ANOVA $P<0.05$ and post hoc Tuckey test $P<0.05$ for human compared to all other species) was reported before by [13].

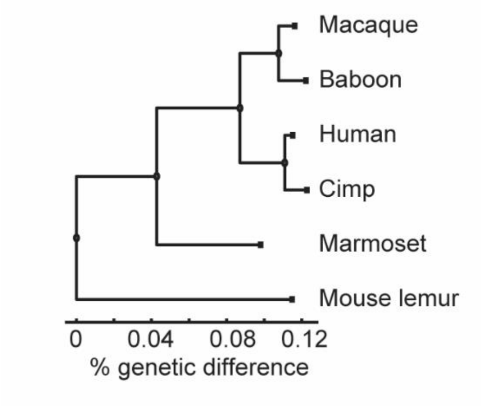

Fig. S2. Normalizing tree. General genetic distance-based phylogenetic tree for the 6 examined species. This tree was used to normalize the sequence distance-based phylogenetic trees produced for each genomic region separately. The normalizing tree was downloaded from http://www.gate.net/~rwms/primegendist.html#Msp where the distances between the nodes were calculated by the PAUP*4.0 software.

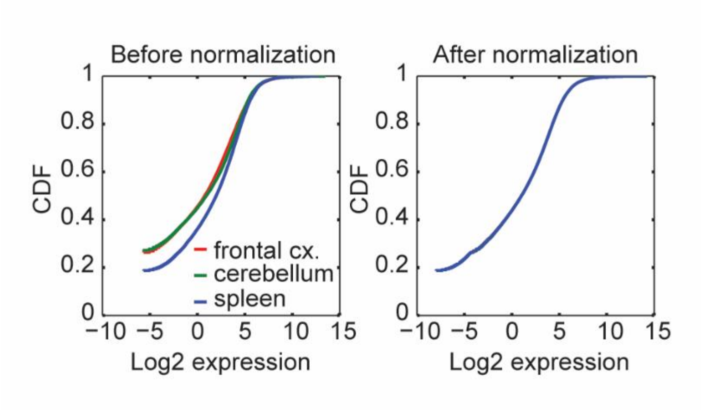

Fig. S3. Quantile normalization of expression data. Cumulative distribution functions (CDF) for three example human tissues; frontal cortex (frontal cx.), cerebellum and spleen, before and after quantile normalization. Note that after normalization distributions of different tissues overlap. In the right graph, the red and green curves are behind the blue curve.

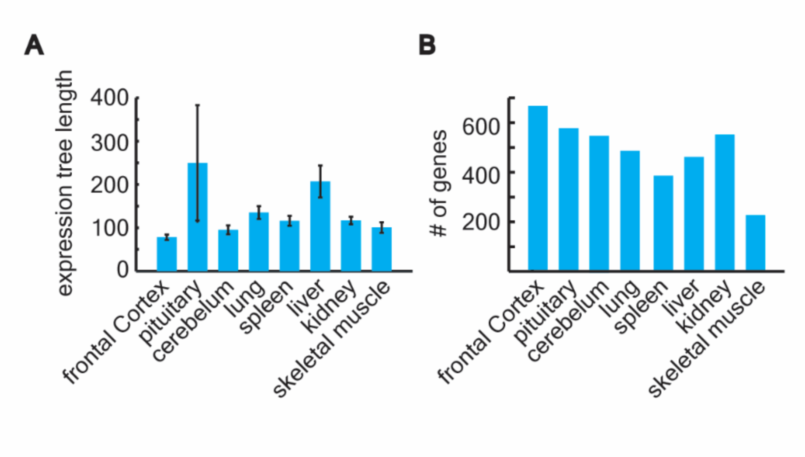

Fig. S4. (**A**) Total length of expression-based trees for each tissue (average and s.e.m). The smaller expression distance between primates for brain regions was reported before by [7,8]. (**B**) Number of genes analyzes in each tissue, after filtering out gene regions with unidentified nucleotides in any species and non-expressed genes.
